# Post-translational modifications in sepsis-induced acute kidney injury: mechanisms and perspectives

**DOI:** 10.3389/fphar.2025.1625139

**Published:** 2025-09-08

**Authors:** Lin Song, Wei Jiang, Ke Liu, Jing Wang, Weilei Gong, Jiangquan Yu, Ruiqiang Zheng

**Affiliations:** ^1^ Northern Jiangsu People’s Hospital Affiliated to Yangzhou University, Yangzhou, China; ^2^ Intensive Care Unit, Northern Jiangsu People’s Hospital, Yangzhou, China; ^3^ Yangzhou University Hospital, Yangzhou, China; ^4^ School of Pharmaceutical Sciences & Institute of Materia Medica, Shandong First Medical University & Shandong Academy of Medical Sciences, Jinan, China

**Keywords:** acute kidney injury, sepsis, post-translational modifications, sepsis-induced acute kidney injury, inflammation

## Abstract

Sepsis-induced acute kidney injury (SA-AKI), a grave clinical issue with a high mortality rate, has impacted millions of individuals worldwide. Sepsis is a syndrome characterized by life-threatening organ dysfunction resulting from a dysregulated host response to infection. Post-translational modifications (PTMs) refer to the chemical alterations that proteins undergo after their synthesis is complete. Various types of PTMs, including phosphorylation, methylation, acetylation, lactylation, and ubiquitination, may play a crucial role in the acute kidney injury (AKI) associated with sepsis by modulating protein function, stability, and interactions. This article highlights the advances in understanding the role of PTMs in the pathogenesis of sepsis-induced acute kidney injury (SA-AKI), addressing existing challenges and offering future perspectives.

## Introduction

The Human Genome Project revealed that the human genome contains approximately 20,000 to 25,000 genes. However, due to alternative splicing, metabolic processes, and post-translational modifications (PTMs), the human proteome comprises over one million distinct proteins (International Human Genome Sequencing Consortium, 2004; Bonasio et al., 2010). PTMs refer to chemical modifications that occur on amino acid residues of proteins following translation, achieved by the addition or removal of specific groups. These modifications regulate protein activity, localization, folding, and interactions with other biomolecules (Bludau and Aebersold, 2020; Chandramouli and Qian, 2009; Pérez-Pérez et al., 2017). To date, with the continuous advancement of high-throughput omics technologies and highly sensitive mass spectrometry techniques, over 600 types of PTMs have been identified (Yuditskaya et al., 2010). The most common PTMs include protein phosphorylation, methylation, acetylation, ubiquitination, and glycosylation. Additionally, a series of novel acylation modifications discovered in recent years, such as lactylation, crotonylation, succinylation, and 2-hydroxyisobutyrylation, have emerged as popular research topics (Biggar and Li, 2015; Kouzarides, 2007; Zhang et al., 2019). These PTMs expand the functionality and diversity of proteins, thereby increasing the complexity of the proteome, which is essential for maintaining cellular vitality and biological processes. PTMs research carries particular significance for understanding diseases such as cardiovascular disorders, cancer, and inflammatory conditions.

**FIGURE 1:**
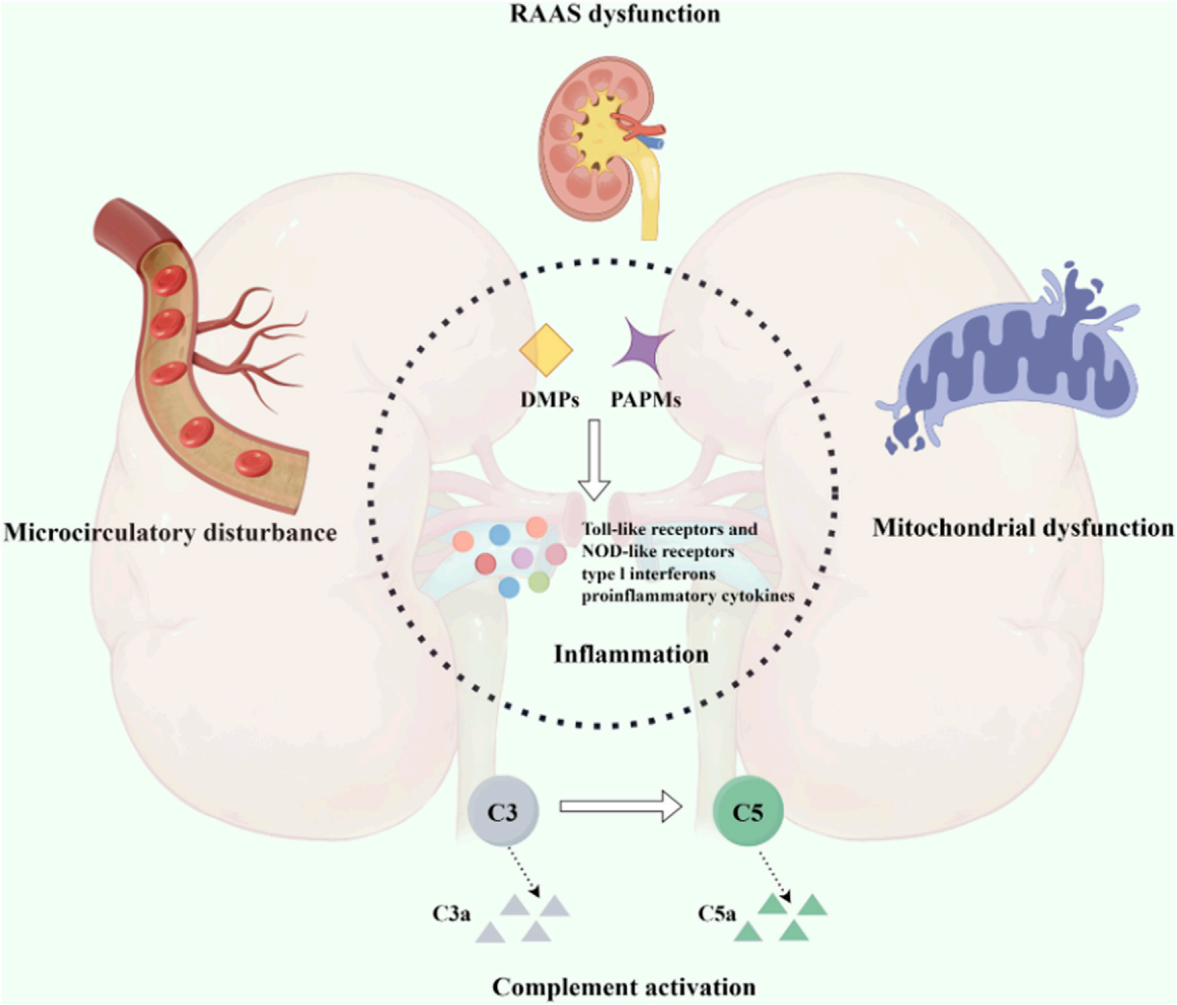
Pathogenesis associated with sepsis-induced acute kidney injury. An intricate interplay among inflammation, complement activation, mitochondrial dysfunction, and microcirculatory impairment.

Sepsis is a life-threatening organ dysfunction caused by a dysregulated host response to infection, and sepsis-induced acute kidney injury (SA-AKI) is a common and serious complication among patients, characterized by an exceedingly high incidence and mortality rate (Remuzzi and Horton, 2013; Shankar-Hari et al., 2016; Peerapornratana et al., 2019). The pathophysiological of SA-AKI involves microvascular damage, which can lead to constriction of the afferent arterioles and increased intratubular pressure, resulting in a sustained decline in glomerular filtration function (Pais et al., 2024). Prolonged sepsis may promote SA-AKI as the dominant renal pathology through microvascular dysfunction, mitochondrial damage, and tubular injury (Kuwabara et al., 2022). Therefore, SA-AKI is associated with poorer outcomes compared to any syndrome within isolated treatment settings. It is linked to longer intensive care units (ICU) and hospital stays in both adults and children, higher mortality rates, increased long-term disability rates, and reduced quality of life (Bagshaw et al., 2007; Program to Improve Care in Acute Renal Disease PICARD Study Group et al., 2011). Although the pathophysiological of SA-AKI have not been fully elucidated, current research predominantly focuses on inflammation, complement activation, mitochondrial dysfunction, and microcirculatory disturbances (Zarbock et al., 2023; Shum et al., 2016) (Figure 1).

The specific mechanisms underlying SA-AKI remain poorly understood, making its treatment a persistent challenge in clinical practice. As an emerging therapeutic target, PTMs has garnered widespread attention and is considered a promising strategy to protect the kidneys and promote renal function repair and recovery. This article provides an in-depth summary and discussion of PTMs associated with the pathogenesis of SA-AKI, including phosphorylation, methylation, acetylation, ubiquitination, and lactylation. These insights may establish a new theoretical framework and therapeutic targets for the treatment of SA-AKI.

## The relationship between phosphorylation and SA-AKI

Phosphorylation of proteins, catalyzed by protein kinases, represents a widespread PTMs that plays a pivotal role in the onset and progression of SA-AKI (Song L. et al., 2024). This modification refers to the attachment of phosphate groups to proteins, primarily at serine, threonine, and tyrosine residues dynamically controlling protein function and localization by activating or inactivating numerous enzymes and receptors through phosphorylation and dephosphorylation, serving as a significant cellular regulatory mechanism (Kamacioglu et al., 2021; Ardito et al., 2017; Wang et al., 2019). For instance, Wnt-5a activates the calcineurin signaling pathway, leading to the dephosphorylation of Nuclear Factor of activated T cells (NFAT) transcription factors and promoting the transcription and phosphorylation of aquaporin 2 (AQP2). This process ultimately ameliorates the downregulation of renal AQP2 induced by sepsis (Wang Y. et al., 2025). In the context of SA-AKI, phosphorylation events can influence the pathophysiology of the disease by modulating inflammatory responses and cell death processes.

**FIGURE 2:**
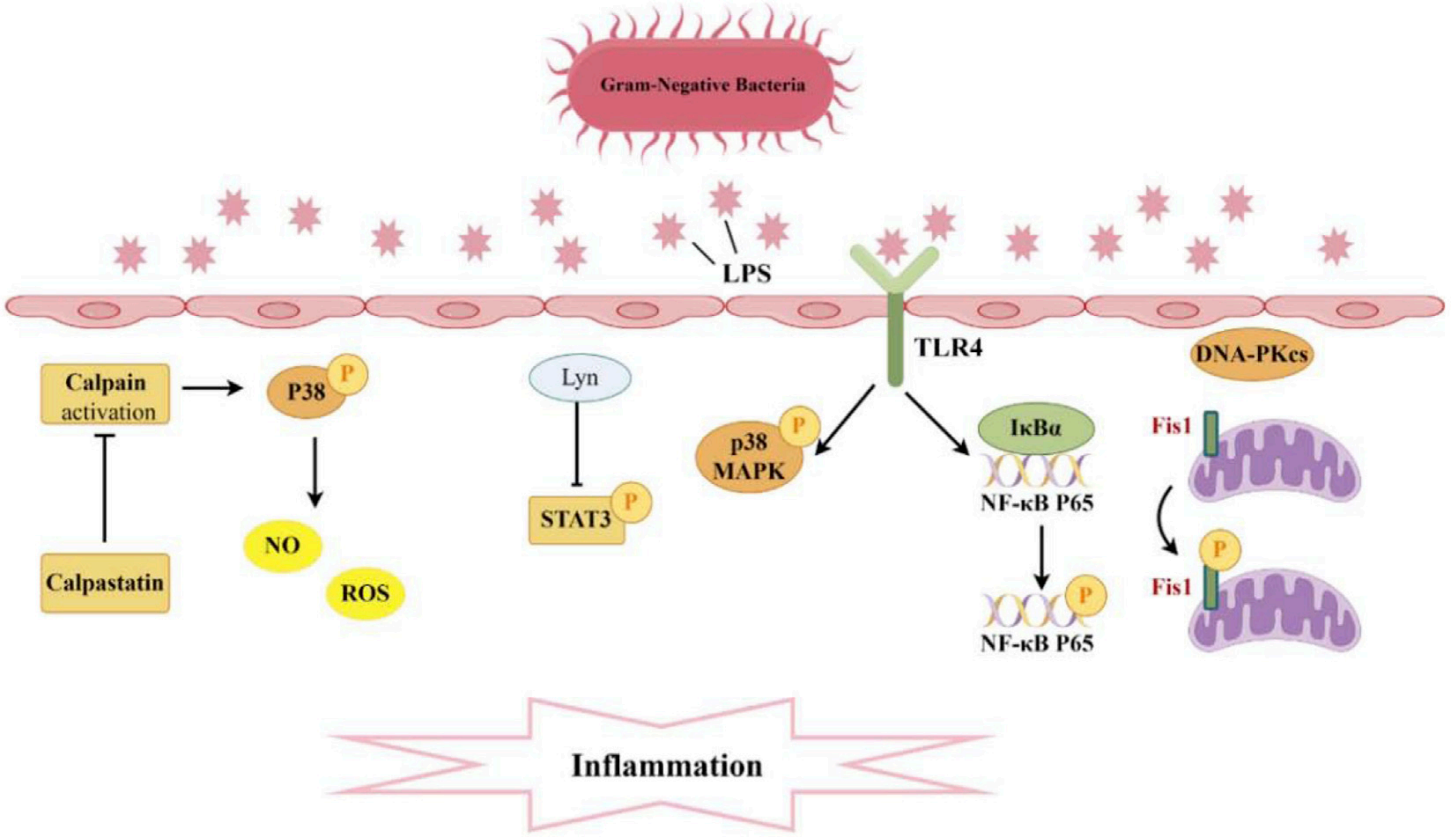
Pathogenesis associated with Phosphorylation and sepsis-induced acute kidney injury. A significant production of inflammatory cytokines occurs within the glomeruli and renal tubular interstitium. The interaction between TLR4 and LPS activates the phosphorylation of P38 MAPK and NF-κB, while Lyn inhibits the phosphorylation of STAT3, thereby diminishing levels of inflammatory mediators. Moreover, the suppression of calpain activation can curtail P38 phosphorylation, reduce ROS, and consequently mitigate endothelial cell apoptosis. Additionally, DNA-PKcs can induce the phosphorylation of Fis1, resulting in mitochondrial dysfunction and subsequent cell apoptosis.

The systemic inflammatory response syndrome induced by infection characterizes sepsis (for the International Sepsis Definitions Conference et al., 2003). Uncontrolled inflammatory mediators bind to pattern recognition receptors on the surfaces of immune cells, endothelial cells, and renal tubular epithelial cells, triggering the substantial release of pro-inflammatory factors that contribute to the development of SA-AKI (Rello et al., 2017). Previous studies have reported a significant increase in the number of macrophages and neutrophils in the glomeruli and renal tubular interstitium of patients with SA-AKI, accompanied by the production of abundant inflammatory cytokines (Aslan et al., 2018; Lerolle et al., 2010). In sepsis animal models, inflammatory factors such as IL-1β and TNF-α are significantly elevated in both serum and kidney, contributing to the occurrence and progression of AKI (Lousa et al., 2022). Lyn, a member of the Src family kinases (SFKs), can reduce the levels of inflammatory mediators by inhibiting the phosphorylation of signal transducer and activator of transcription 3 (STAT3) and apoptosis, exerting a protective effect on renal tubular injury in SA-AKI mice (Thomas and Brugge, 1997; Wang et al., 2017; Schreiner et al., 2002) (Figure 2). Treatment with the tyrosine-protein kinase (Lyn) agonist MLR-1023 further demonstrates improvement in kidney function, suppression of STAT3 phosphorylation, and reduction of apoptosis (Li N. et al., 2023). The elevated phosphorylation level of stigmasterol (STIG) may represent a crucial pathological mechanism in SA-AKI. Research has revealed that the Cytosolic mtDNA-cGAS-STING signaling axis can activate the LRP3 inflammasome by promoting the phosphorylation of STIG, TANK-binding kinase 1 (TBK1), and interferon regulatory factor 3 (IRF3), thereby exerting a proinflammatory effect in SA-AKI. These mechanistic insights suggest phosphorylation-targeted interventions could therapeutically modulate SA-AKI inflammatory responses (Luo et al., 2024).

Toll-like receptors (TLRs) on the surface of immune cells serve as the first line of defense, detecting invading microbes and initiating the innate immune response to recognize and eliminate pathogens. Toll-like receptor 4 (TLR4) serves as a receptor for the lipopolysaccharides (LPS) or endotoxins of Gram-negative bacteria (Moresco et al., 2011). There is evidence to suggest that TLR4 is involved in the inflammation and renal injury associated with LPS-induced AKI. In mice with AKI, TLR4 expression is upregulated, leading to the activation and phosphorylation of p38 mitogen-activated protein kinase (MAPK), which mediates the pyroptotic response. Silencing TLR4 inhibits the phosphorylation of p38 MAPK via the myeloid differentiation primary response gene 88 (MyD88)/TIR domain-containing adapter-inducing interferon-β (TRIF) pathway, thereby reducing the levels of pro-inflammatory factors. This results in a decreased inflammatory response in AKI mice, alleviating renal injury and exerting a protective effect (Yue et al., 2023) (Figure 2). On the other hand, the binding of TLR4 to LPS also activates nuclear factor kappa-B (NF-κB). It is well known that the genes encoding many pro-inflammatory cytokines are regulated by NF-κB transcription factors (Kaplan et al., 2014). This pathway plays a crucial role in mediating the inflammatory response during, further contributing to kidney injury. Research has found that the novel natural product, Paeonol and Fortunellin, can dose-dependently reduce LPS-induced pro-inflammatory cytokines while increasing anti-inflammatory cytokines. It achieves this by inhibiting the expression of phosphorylated NF-κB p65, inhibitor of nuclear factor κB-α (IκBα), and inhibitor of Nuclear Factor-κB kinase β (IKKβ) in dendritic cells, as well as suppressing TLR4 expression and the DNA-binding activity of NF-κB p65. Therefore, Paeonol holds potential therapeutic value in the treatment of SA-AKI (Fan et al., 2016; Liu T. et al., 2024). In addition to Paeonol, embelin has been shown to alleviate renal dysfunction and pathological kidney damage in LPS-induced sepsis mouse models. It does this by inhibiting the translocation of phosphorylated NF-κB p65, thereby suppressing the activation of M1 macrophages and reducing the secretion of inflammatory factors (Tang et al., 2023). Ursodeoxycholic acid can activate the nuclear factor erythroid 2-related factor 2 (Nrf2)/heme oxygenase 1 (HO-1) pathway by promoting the phosphorylation and nuclear translocation of Nrf2, while inhibiting the phosphorylation and nuclear translocation of the NF-κB p65 subunit, thereby blocking the NF-κB pathway and reducing oxidative stress and inflammatory responses (Lou et al., 2025). The promotional effect of the immunoproteasome subunit PSMB8 in septic AKI is associated with the activation of the NF-κB pathway. Knocking down proteasome subunit beta type-8 (PSMB8) can inhibit the phosphorylation and degradation of IκBα, blocking the NF-κB pathway and thus reducing kidney inflammation and damage caused by sepsis. Dihydromyricetin nanoparticles downregulate the expression of downstream inflammatory factors and apoptotic effectors by inhibiting the expression, phosphorylation, and nuclear translocation of TLR4, MyD88, and NF-κB p65 (Yin et al., 2024). These findings deepen our understanding of the molecular mechanisms underlying septic kidney injury and provide new ideas and drug intervention targets for the treatment of septic acute kidney injury.

Vascular endothelial cells renal endothelial barrier and homeostasis and integrity, with endothelial dysfunction emerging as a key factor in AKI pathogenesis. This dysfunction can lead to increased vascular permeability, inflammation, and ultimately contribute to renal damage during sepsis (Bonventre and Yang, 2011). Addressing endothelial dysfunction may represent a potential therapeutic strategy for mitigating AKI in sepsis patients. Calpain is a calcium-dependent cysteine protease, and its activation mediates endothelial cell apoptosis, which can compromise vascular integrity. The calcium-dependent cysteine protease calpain mediates endothelial cell apoptosis when activated, disrupting vascular integrity (Saez et al., 2006; Zhang et al., 2017). The dysregulation of calpain activity in conditions such as sepsis may play a significant role in the development of AKI by exacerbating endothelial dysfunction and promoting further renal damage. Research has revealed that endothelial-specific calpain small subunit 4 (Capn4) knockout and calpastatin overexpression, an endogenous calpain inhibitor, can alleviate renal dysfunction in mice subjected to endotoxemia. Moreover, knockout of calpain in endothelial cells may confer protective effects in LPS-induced AKI by inhibiting p38 phosphorylation and diminishing the expression of inducible nitric oxide synthase (iNOS), and further decreasing endothelial cell apoptosis caused by excessive NO/ROS production (Liu et al., 2020) (Figure 2).

The pathogenesis of AKI is primarily driven by the death of renal tubular cells, independent of triggering factors (Sancho-Martínez et al., 2015). Genomic integrity is vital for cell survival under stress, and the catalytic subunit of DNA-dependent protein kinase (DNA-PKcs) a key DNA repair enzyme—showing elevated expression during apoptosis. Although DNA-PKcs is typically localized in the nucleus, its presence in the cytoplasm has been detected in both mouse renal tissues and urinary sediment from patients with AKI, and its increased amount correlates with renal dysfunction (Dynan and Yoo, 1998; Li et al., 2013). Both *in vivo* and *in vitro* experiments have demonstrated that cytoplasmic DNA-PKcs can induce the phosphorylation of fission 1 protein (Fis1) at the Thr34 site. This phosphorylation event enhances the association between Fis1 and dynamin-Related Protein 1 (Drp1), leading to mitochondrial fragmentation and apoptosis of renal tubular cells, which ultimately contributes to the decline in renal function during AKI (Wang et al., 2022) (Figure 2). Currently, clinical trials on DNA-PKcs inhibitors are underway, presenting a potential approach for the prevention of SA-AKI. In a study, it was found that H_2_S not only inhibited the expression of protein kinase R-like endoplasmic reticulum kinase (PERK) but also prevented its phosphorylation process, thereby upregulating while downregulating Bax. This series of changes effectively alleviated sepsis-induced renal cell apoptosis and tissue damage (Song C. et al., 2024). Another study pointed out that wild-type p53-induced phosphatase 1 (WIP1), through dephosphorylating p38 MAPK, further inhibited the activation of NOD-like receptor family pyrin domain containing 3 (NLRP3) inflammasome and caspase-1, reducing the occurrence of pyroptosis in renal tubular epithelial cells. This study emphasized that the downregulation of WIP1 expression and the continuous phosphorylation activation of p38 MAPK may be one of the important pathological mechanisms of SA-AKI (Wang et al., 2024a). Additionally, MicroRNA-17-5p has also been proven to activate the downstream Akt signaling pathway by inhibiting the expression and phosphorylation of specific proteins, thereby reducing sepsis-induced renal inflammation and cell apoptosis (Sun J. et al., 2024).

In recent years, research has unveiled that metabolic reprogramming plays a pivotal role in the immune response of the host to infection, representing a novel target for inflammatory diseases (Gómez et al., 2017). Metabolomic analysis of renal tissue in a cecal ligation and puncture (CLP) sepsis mouse model showed significant accumulation of glycolytic intermediates (Waltz et al., 2016). Smith et al. demonstrated that following LPS induction, the metabolic profile of mouse renal tissue shifts toward aerobic glycolysis, which is associated with a decline in renal function (Smith et al., 2014). Conversely, inhibition of aerobic glycolysis can enhance survival rates and protect sepsis mice from kidney injury (Tan et al., 2020). Additionally, studies have indicated a reciprocal influence between autophagy and metabolism. In both septic mice and LPS-treated HK-2 cells, treatment with the glycolysis inhibitor 2-DG increased the expression of sirtuin 3 (SIRT3) and phosphorylated AMP-activated protein kinase (p-AMPK), promoting autophagy in tubular epithelial cells and attenuating apoptosis. However, the glycolytic metabolite lactate can downregulate the expression of SIRT3 and p-AMPK, thereby inhibiting autophagy in LPS-treated HK-2 cells and enhancing apoptosis. Further research has shown that the pharmacological blockade of autophagy using 3-methyladenine (3-MA) partially eliminates the protective effects of 2-DG against AKI (Tan et al., 2021). Thus, The early metabolic reprogramming induced by AKI not only protects the kidneys from further damage but also determines the fate of tissue repair and the progression of fibrosis and chronic organ dysfunction (Gómez et al., 2017; Zager, 2013).

In the past decade, extensive research has been conducted to discover and validate new biomarkers for AKI. Quantitative proteomic analyses have identified phosphorylated myosin regulatory light chain 12B (MYL12B) as a potential biomarker associated with the progression and development of SA-AKI. Plasma levels of both MYL12B and its phosphorylated form increase significantly in SA-AKI patients but show no alteration in the control group (Hasson et al., 2021). The potential of using this biomarker for screening and treating SA-AKI warrants further investigation.

## The relationship between ubiquitination and SA-AKI

Ubiquitin, a low-molecular-weight protein consisting of amino acid residues, is expressed in almost all eukaryotic cells and functions as a post-translational marker for protein degradation. Ubiquitination refers to the process of attaching small ubiquitin proteins to target proteins, regulating their stability, activity, and interactions through this covalent modification (Varshavsky, 2017; Pohl and Dikic, 2019). Currently, there is a growing body of evidence suggesting that ubiquitination is involved in the development and progression of sepsis.

**FIGURE 3:**
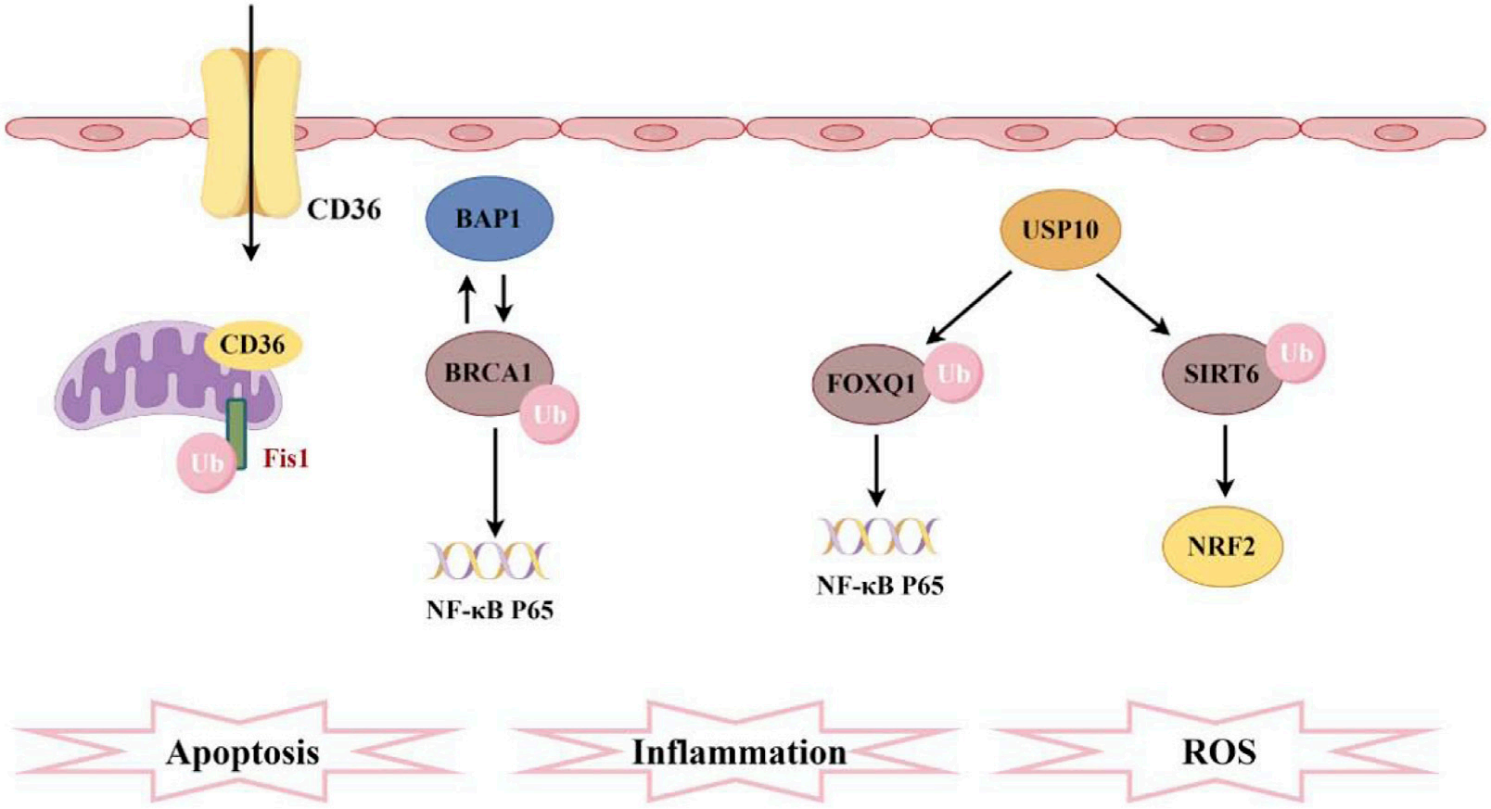
Pathogenesis associated with ubiquitination and sepsis-induced acute kidney injury. CD36 promotes ferroptosis in proximal tubular cells by regulating the ubiquitination of FSP1. The interaction between BAP1 and BRCA1 enhances the stability of BRCA1 protein through deubiquitination, thereby inhibiting NF-κB. Furthermore, FOXQ1, deubiquitinated by USP10, ameliorates cellular inflammation and apoptosis. Additionally, USP10 interacts with SIRT6 to suppress its ubiquitination, alleviating oxidative stress.

Recently, Liao Xiaohui’s team provided the first evidence of ferroptosis suppressor protein 1 (FSP1) ubiquitination in SA-AKI. Their study demonstrated that the scavenger receptor CD36 promotes the accumulation of oxylipins and ferroptosis in renal tubular cells by regulating the ubiquitination of FSP1 at K16 and K24 sites in proximal tubular cells, leading to the progression of AKI (Ma et al., 2024) (Figure 3). It is noteworthy that, unlike typical ubiquitination sites, this study provides evidence of ubiquitination occurring at lysine residues that have previously been reported as ubiquitination sites in viral infections and the immune system. This suggests that PTMs occurring at different critical lysine residues may provide a potential explanation for the functional heterogeneity observed among ubiquitinated proteins. Additionally, previous studies on CD36 in kidney injury have primarily focused on metabolic inflammation, energetic reprogramming, apoptosis, and fibrosis (Shu et al., 2022; Yang et al., 2017). This research is the first to evaluate the relationship between CD36 and AKI, revealing a significant increase in CD36 expression in the renal tissues of patients with AKI. Elevated levels of CD36 may serve as an independent correlate of renal function in patients with AKI. The ubiquitin-like protein (UBL) family shares key structural features with ubiquitin and modifies substrates involved in cellular proliferation and metabolism, thereby mediating inflammation and apoptosis in chronic kidney disease (Saifee and Zheng, 2008; Herrmann et al., 2007; Gong et al., 2010). Previous studies have shown that ubiquitin-like protein 4A (UBL4A) promotes the secretion of inflammatory cytokines, and knocking down UBL4A provides protective effects against inflammatory diseases. Furthermore, overexpression of miR-34b-3p can alleviate kidney injury in sepsis mice by downregulating the UBL4A/NF-κB pathway (Liu C. et al., 2019). In fact, the ubiquitin-proteasome system mediates ubiquitin-like protein (UBL) coupling to maintain cellular protein homeostasis, and this system can regulate renal inflammation and influence AKI (Cardozo and Pagano, 2004).

Ubiquitination triggers protein degradation via the proteasomal pathway, leading to the relocalization of proteins, whereas deubiquitination enhances protein stability (Mattiroli and Penengo, 2021; Liu F. et al., 2024). BRCA-1 associated protein-1(BAP1) is a tumor suppressor gene that encodes a deubiquitinating enzyme, mediating various key cellular pathways associated with cell differentiation, the cell cycle, transcription, and DNA damage response (Wang et al., 2016; Louie and Kurzrock, 2020). Research has reported that BAP1 interacts with breast cancer 1 (BRCA1) and enhances the stability of the BRCA1 protein through deubiquitination modifications (Dkhissi et al., 2015). This interaction further inhibits the NF-κB signaling pathway, thereby protecting mice from AKI induced by sepsis (Figure 3). Ubiquitin carboxyl-terminal hydrolase 10 (USP10) is also a deubiquitinating enzyme, with forkhead box Q1 (FOXQ1) identified as one of its substrates. Upregulation of USP10 can alleviate LPS-induced cell apoptosis and inflammatory responses, while inhibition of FOXQ1 exacerbates these effects (Luo et al., 2023). During AKI, activation of the ubiquitination pathway accelerates the breakdown of inflammation- and apoptosis-related proteins, directly influencing cell fate (Wang and Robbins, 2014). USP10-mediated deubiquitination of FOXQ1 ameliorates kidney injury in sepsis mice induced by CLP and LPS by targeting the CREB5/NF-κB axis, thereby alleviating inflammation and apoptosis in HK-2 cells (Zhao et al., 2024). USP10 can also interact with SIRT6, inhibiting its ubiquitination and degradation, thereby preventing fatty liver and cardiac hypertrophy. In a mouse model of AKI, USP10 alleviates sepsis-induced oxidative stress in renal tissue by enhancing the stability of SIRT6 and activating the NRF2/ARE signaling pathway (Gao et al., 2021) (Figure 3). Recent research highlights the deubiquitinase Ubiquitin-specific protease 9X (Usp9x) in the development of SA-AKI. Usp9x affects the TLR4/NF-κB pathway, which is a key signaling pathway for inflammatory and immune responses. The study found that Usp9x interacts with TLR4, leading to its upregulation through deubiquitination. This stabilization exacerbates inflammation and renal tubular epithelial cell apoptosis, accelerating SA-AKI progression (Gong et al., 2024). Ubiquitination occurs through a series of enzymatic reactions that require the sequential action of three types of enzymes: E1 ubiquitin-activating enzymes, E2 ubiquitin-conjugating enzymes, and classic E3 ubiquitin ligases. E3 ubiquitin ligases can be primarily classified into three types: Really Interesting New Gene (RING) type, Homologous to E6AP C-terminus (HECT) type, and RING-between-RING (RBR) type. The specific recognition of substrates by these E3 ligases makes them particularly important in the ubiquitination pathway, and they have been extensively studied in various diseases (Dewson et al., 2023; Brillada and Trujillo, 2023). However, their mechanisms of action in SA-AKI are still not well understood. Although circular RNA ITCH (CircITCH) is a broad-spectrum tumor-suppressive circular RNA, its encoding gene ITCH (E3 ubiquitin ligase) has been found to reduce renal cell apoptosis, alleviate oxidative stress markers, and decrease levels of inflammatory factors (Han et al., 2021; Su et al., 2022). However, this study did not investigate the role of E3 ubiquitin ligase and ubiquitination modifications in these processes. This suggests that future research will continue to expand this field, providing more insights into the relationship between ubiquitination and SA-AKI. As science continues to evolve, a deeper exploration of the molecular mechanisms of ubiquitination will offer new perspectives for a more profound understanding of the pathological processes underlying SA-AKI, while also providing more precise targets for future therapeutic strategies.

## The relationship between acetylation and SA-AKI

**FIGURE 4:**
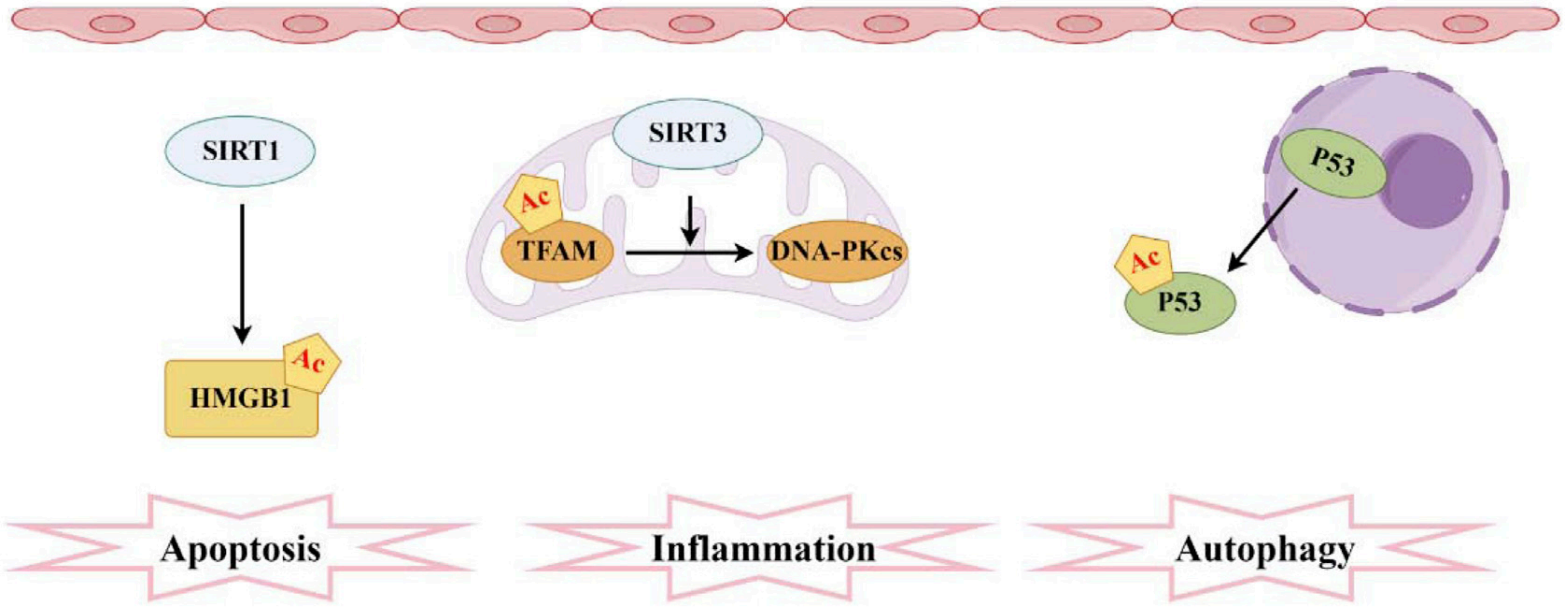
Pathogenesis associated with acetylation and sepsis-induced acute kidney injury. The sirtuin family comprises the most prevalent deacetylases, with SIRT1 mediating the acetylation of HMGB1 and SIRT3 facilitating the acetylation of TFAM. Moreover, elevated levels of acetylated P53 in RTECs hinder autophagy.

Protein acetylation is the process of transferring an acetyl group to the side chain of amino acids. As a significant PTMs, it plays a crucial role in regulating protein function during cellular stress and metabolic processes. Histone acetylation is the most prevalent form of acetylation, regulated by two types of enzymes: histone acetyltransferases (HATs) and histone deacetylases (HDACs) (Marmorstein and Zhou, 2014). HATs can be classified into four major categories based on their structure and properties: CBP/p300, GCN5, MYST, and SRC/p160 (Seto and Yoshida, 2014). The NAD^+^-dependent sirtuin (SIRT) family constitutes the most abundant class of deacetylases (Wu et al., 2022). Resveratrol, a known SIRT1 activator, effectively restore the activity of SIRT1/3 in sepsis rats with AKI, reduce the acetylation levels of recombinant superoxide dismutase 2 (SOD2), improve oxidative stress and mitochondrial function in renal tubular epithelial cells, and extend survival time (Xu et al., 2016). Similarly, SIRT2, another member of the sirtuins family, is involved in regulating autophagy in SA-AKI. Inhibiting SIRT2 can increase the acetylation level of the transcription factor FX1, thereby promoting autophagy and reducing AKI in sepsis mice. Acetylated FX1 binds to the promoter regions of autophagy-related genes, upregulating the transcription of these genes and activating autophagy (Yu et al., 2025). It is noteworthy that the deacetylation of high mobility group protein B1 (HMGB1) mediated by SIRT1 has garnered considerable attention over the past few decades (Wei et al., 2022). The acetylation modification of HMGB1 is a critical process for its translocation from the nucleus to the cytoplasm and subsequent extracellular secretion, thereby accelerating the progression of SA-AKI. However, SIRT1 regulates the deacetylation of HMGB1 in renal cells, thereby inhibiting downstream inflammatory signaling (Wei et al., 2019). Another study found that melatonin can promote the SIRT3-mediated deacetylation of mitochondrial transcription factor A (TFAM), thereby enhancing mitochondrial autophagy flux and mitigating SA-AKI. Given its clinical availability, melatonin demonstrates significant potential for translational application in this context (Deng et al., 2024). Numerous studies have shown that the acetylation and deacetylation of functional proteins play a critical role in apoptosis, inflammatory responses, metabolic adaptation, and impaired microcirculation function in renal tubular epithelial cells (RTECs) (Deng et al., 2021; Sun et al., 2021). Following sepsis stimulation, the autophagy level of RTECs temporarily increases and then drops sharply. Autophagy inhibition is accompanied by an increase in renal tubular injury scores. In contrast, autophagy agonists can reduce renal tubular damage after sepsis (Lee et al., 2022). After sepsis, there is no significant change in the total protein expression of p53 in RTECs, but there is an increase in nuclear-to-cytoplasmic translocation and acetylation. Elevated p53 acetylation levels can promote autophagy inhibition and exacerbate SA-AKI, while SIRT1-induced p53 deacetylation can attenuate this process to some extent (Sun et al., 2021) (Figure 4). There has been corresponding research on histone acetylation in mitochondrial dysfunction of renal tubular epithelial cells induced by LPS. LPS can activate the acetyltransferase P300, promoting the acetylation of histone H3, which in turn upregulates the expression of mitochondrial enzyme 2-Methyltetrahydrofuran (MTHF2). Knocking down MTHF2 can improve mitochondrial dysfunction and cell damage induced by LPS (Hu, 2024). Currently, several studies have thoroughly investigated potential drugs targeting SA-AKI. The ability of HDAC inhibitors (HDACi) to regulate the acetylation of both histone and non-histone proteins has long been considered an effective anti-inflammatory mechanism (von Knethen and Brüne, 2019; Williams et al., 2019). Treatment with pan-HDAC inhibitors and specific subtype-selective HDACi can confer a survival advantage and reduce the expression of pro-inflammatory mediators (Zhao et al., 2013). The protective effects of the class IIa HDAC inhibitor TMP195 on the kidneys have been explored in a mouse model of AKI induced by LPS. TMP195 markedly lowers serum creatinine and blood urea nitrogen levels while attenuating LPS-induced kidney damage. Additionally, it reduces tubular cell apoptosis and inflammatory responses (Zhang et al., 2020). Its potent renal protective effects in SA-AKI suggest that targeting class IIa HDACs could serve as a novel therapeutic strategy for treating SA-AKI, while potentially avoiding the unintended adverse effects associated with pan-HDAC inhibitors. Dexmedetomidine (DEX) is an α-2 adrenergic receptor (α2-AR) agonist that is used as an analgesic and sedative. α2-AR agonists offer a preventive option for the treatment of sepsis (Hayashi et al., 1996; Cetindag Ciltas et al., 2022). The specific mechanism involves DEX primarily reducing the levels of HDAC2 and HDAC5 while increasing acetylated histone H3. This action leads to a decrease in the production of inflammatory cytokines in sepsis mice and enhances survival rates (Hsing et al., 2012). Furthermore, Astragalsi IV has the capability to activate the deacetylase SIRT1, which in turn deacetylates the transcription factor FX3a. This process facilitates the nuclear translocation and transcriptional activity of FX3a, upregulating the expression of antioxidant enzymes and autophagy-related genes (Zha et al., 2024). Consequently, it inhibits pyroptosis of renal tubular epithelial cells induced by sepsis, thereby alleviating AKI. Additionally, Zn^2+^ upregulates the expression of the deacetylase SIRT7, subsequently increasing the acetylation level of the mitochondrial protein Parki. The acetylation of Parki promotes its localization and activity on the outer mitochondrial membrane, thus facilitating the autophagic clearance of damaged mitochondria and improving mitochondrial dysfunction and AKI caused by sepsis (Guo et al., 2024).

Research on SA-AKI has extensively explored phosphorylation, ubiquitination, and acetylation mechanisms. We summarize key findings to clarify their underlying pathways and molecular targets (Table 1).

**TABLE 1:** Mechanisms of phosphorylation, ubiquitination, and acetylation in SA-AKI.

PTMs	Subject	PTMs marker	Model	Expression variation in model	Proposed pathway	Effect of epigenetic	References
Phosphorylation	Lyn	STAT3	CLP Mice, FIP Mice, BUMPT + LPS	↘	Lyn/STAT3	proinflammatory factor ↘, apoptosis↘	Li et al. (2023a)
Phosphorylation	mtDNA	BK-1, IRF3, NF-kB	LPS Mice, HK-2 cells + LPS	↖	mtDNA-cGAS-STING/NLRP3	proinflammatory factor↖, mtDNA↘	Luo et al. (2024)
Phosphorylation	TLR4	p38 MAPK	CLP Mice, HK-2 cells + LPS	↖	TLR4/MyD88/TRIF/p38 MAPK	proinflammatory factor↖, antiinflammatory factor↘	Yue et al., (2023)
Phosphorylation	Paeonol	NF-κB p65	LPS Mice, DC + LPS	↘	TLR4/NF-κB	proinflammatory factor↘, antiinflammatory factor↖	Fan et al. (2016)
Phosphorylation	Fortunellin	P65, IκBα	CLP Mice, HK-2 cells + LPS	↘	Fortunellin/TLR4/NF-κB	antioxidant effects↖, proinflammatory factor↘	Liu et al. (2024a)
Phosphorylation	Embelin	NF-κB p65 at the ser536 site	LPS Mice, HK-2 cells + LPS, BMDM + LPS	↘	NF-κB p65	proinflammatory factor↘, antiinflammatoryfactor↖, macrophage M1 activation↘	Tang et al. (2023)
Phosphorylation	UDCA	NF-κB p65	LPS Mice, HK-2 cells + LPS	↘	UDCA/Nrf2/HO-1/NF-κB	proinflammatory factor↘, oxidative stress↘, apoptosis↘	Lou et al. (2025)
Phosphorylation	Dihydromyricetin	NF-κB p65, IκBα	LPS Mice	↘	CS-DMY-NPs/TLR4/NF-κB p65/IκBα	antioxidant capacity↖, apoptosis↘, proinflammatory factor↘	Yin et al. (2024)
Phosphorylation	calpain	p38	LPS Mice, PMEC + LPS	↘	p38/iNO,NO/ROS	iNOS expression↘, endothelial apoptosis↘, NO/ROS production↘	Liu et al. (2020)
Phosphorylation	DNA-PKcs	Fis1	LPS Mice, HK-2 cells + LPS	↖	DNA-PKcs/Fis1/Drp1	mitochondrial fragmentation↖	Wang et al. (2022)
Phosphorylation	H_2_S	PERK	LPS Mice	↖	H_2_S/PERK/Bax-Bcl2	Endoplasmic reticulum stress↖	Song et al. (2024b)
Phosphorylation	WIP1	p38 MAPK	LPS Mice, HK-2 cells + LPS	↖	WIP1/p38 MAPK	pyroptosis↘	Wang et al. (2024a)
Phosphorylation	MicroRNA-17-5p	Smad3	LPS Mice	↘	TGFβR2/TGF-β/Smad3	apoptosis↘, proinflammatory factor↘	Sun et al. (2024a)
Phosphorylation	2-DG	ERK, p38	CLP Mice, Neutrophils + LPS	P38↖, ERK↘	2-DG/GRK2/CXCR2	survival rate↖, bacterial clearance↖	Tan et al. (2020)
Phosphorylation	Lactate	AMPK	CLP Mice, HK-2 cells + LPS	↘	lactate/Sirtuin 3/AMPK	autophagy↘, apoptosis↖	Tan et al. (2021)
Ubiquitination	BRCA1	BAP1	CLP Mice, RTECs + LPS	↖	BAP1/BRCA1/NF-κB	apoptosis↘, proinflammatory factor↘	Luo et al. (2023)
Ubiquitination	USP10	FOXQ1	CLP Mice, HK-2 cells + LPS	↘	FOXQ1/CREB5/NF-κB	apoptosis↘, inflammatory response↘	Zhao et al. (2024)
Ubiquitination	USP10	USP10	CLP Mice, HK-2 cells + LPS	↘	USP10/SIRT6/NRF2/ARE	apoptosis↖, oxidative stress↖	Gao et al. (2021)
Ubiquitination	Usp9X	TLR4	CLP Rats, NRK52E + LPS	↖	Usp9x/TLR4/nf-κb	inflammation↖, apoptosis↖	Gong et al. (2024)
Acetylation	Resveratrol	SOD2	CLP Rats, RTECs + LPS	↘	SIRT3/SOD2	oxidative stress↘, mitochondrial function↖	Xu et al. (2016)
Acetylation	AGK2	FOXO1	CLP Mice, HK-2 cells + LPS	↖	SIRT2/FOXO1	autophagy↖	Yu et al. (2025)
Acetylation	SIRT1	HMGB1	CLP Mice, HK-2 cells + LPS	↖	SIRT1/HMGB1	inflammatory signaling↘	Wei et al. (2019)
Acetylation	Melatonin	TFAM	CLP Mice, HK-2 cells + LPS	↘	SIRT3/TFAM-K154	mitophagy↖	Deng et al. (2024)
Acetylation	SIRT1	Beclin1	CLP Mice, HK-2 cells + LPS	↖	SIRT1/Beclin1	autophagy↖	Deng et al. (2021)
Acetylation	SIRT1	P53	CLP Mice, HK-2 cells + LPS	↖	SIRT1/P53	autophagy↖	Sun et al. (2021)
Acetylation	EP300	H3	NRK52E cells + LPS	↘	EP300/H3/MTHFD2	apoptosis↘, mitochondrial dysfunction↘	Hu (2024)
Acetylation	TMP195	H3	LPS Mice, TKPT cells + LPS	↖	TMP195/HDAC4/H3	apoptosis↘, inflammation↘	Zhang et al. (2020)
Acetylation	Dexmedetomidine	H3	CLP Mice, NRK52E cells + LPS	↖	Dex/BMP-7/HDAC2/HDAC5	proinflammatory factor↘	Hsing et al. (2012)
Acetylation	Astragaloside IV	FOXO3a	LPS Rats, HK-2 cells + LPS	↘	AS-IV/SIRT1/FOXO3a	pyroptosis↘, proinflammatory factor↘	Zha et al. (2024)
Acetylation	Zn^2+^	Parkin	CLP Rats, HK-2 cells + LPS	↖	Zn^2+^/SIRT7/Parkin	mitophagy↖, NLRP3 inflammasome activation↘, apoptosis↘	Guo et al. (2024)

## The relationship between lactylation and SA-AKI

Lactate, traditionally regarded as a mere metabolic waste product and the final byproduct of glycolysis, is increasingly recognized as a fundamental participant in energy metabolism rather than just a transient metabolite under hypoxic conditions (Rabinowitz and Enerbäck, 2020). This circulating metabolite mediates energy exchange while exhibiting anti-inflammatory, immune-regulatory, and tissue-repair functions through both intracellular and intercellular shuttling (Brooks, 2018). In recent years, the alterations in energy metabolism associated with septic patients have increasingly become a focal point of research interest (Cheng et al., 2016). Transcriptomic and metabolic profiling reveal that the transition from oxidative phosphorylation to aerobic glycolysis constitutes a critical element in early host defense activation during sepsis (Shankar-Hari et al., 2016). Furthermore, the glycolytic product lactate serves as a significant indicator of sepsis severity and mortality. An increase in lactate production coupled with a decrease in lactate clearance leads to the accumulation of lactate in septic patients (Rabinowitz and Enerbäck, 2020). Clinical studies consistently identify serum lactate as a prognostic biomarker, where sustained elevation strongly correlates with sepsis-related mortality (Pan et al., 2019; Sacha et al., 2022; Hernandez et al., 2019).

**FIGURE 5:**
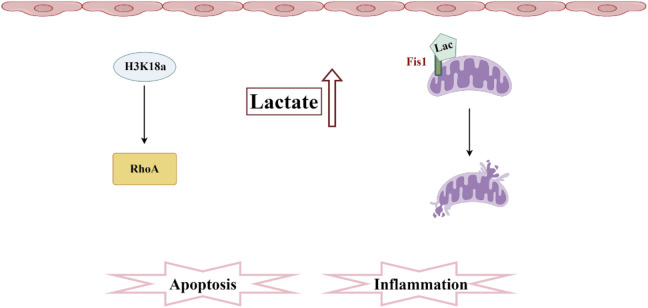
Pathogenesis associated with lactylation and sepsis-induced acute kidney injury. In SA-AKI, elevated levels of lactate and histone lactylation, particularly the increased lactylation of H3K18, activate RhoA protein, thereby triggering inflammation and apoptosis. Additionally, lactate mediates the lactylation of Fis1, promoting mitochondrial fission and exacerbating cellular apoptosis.

In 2019, Professor Zhao Yingmin’s team discovered a novel acylation modification—lactylation—and demonstrated that lactate can directly promote the lactylation of histones, thereby regulating gene expression (Zhang et al., 2019). This discovery bridges glycolysis with epigenetic regulation, opening new avenues for research into sepsis. Due to the metabolic reprogramming of RTECs in the early stages of SA-AKI, there may be abnormalities in the lactylation of histones within these cells (Kaushik et al., 2019). Research has found that histone lactylation levels are elevated in SA-AKI, partly mediated by the regulation of gene expression through histone lactylation to modulate cellular metabolism (Xie et al., 2016). This suggests that correcting abnormal lactate levels could effectively protect renal function from damage. SA-AKI promotes an increase in lactate and H3K18 lactylation levels, activating the expression of RhoA protein, which mediates downstream inflammation and apoptosis, contributing to renal injury. This research reveals a new mechanism linking lactate and organ damage in SA-AKI, emphasizing the importance of therapeutic strategies aimed at lowering serum lactate levels in septic patients. Recent research has discovered that in a septic mouse model, the lactylation levels of H3K18 and Ezrin in kidney tissue are significantly elevated, and these levels positively correlate with the degree of kidney function impairment. The lactylation of H3K18 and Ezrin can upregulate the expression of pro-inflammatory genes, playing a pivotal role in SA-AKI (Qiao et al., 2024). Another study confirmed that both the increase of endogenous lactate and the supplementation of exogenous lactate can further mediate the lactylation level of Fis1 in renal tubular epithelial cells, ultimately promoting excessive mitochondrial fission and exacerbating apoptosis in SA-AKI. Reducing lactate levels and Fis1 lactylation can improve tubular epithelial cell injury and alleviate SA-AKI (An et al., 2023) (Figure 5).

The research on lactylation in sepsis is still in its infancy, but it has already shown great potential. In addition to the aforementioned studies, lactylation of the non-histone protein HMGB1 derived from macrophages in sepsis has also been reported (Zhu et al., 2024). Elucidating lactylation’s role in inflammation, multiorgan dysfunction, and immune regulation may advance sepsis diagnosis, treatment, and prognostic assessment. Lactylation could potentially supplant lactate as a more robust molecular marker for clinical decision-making.

## The relationship between methylation and SA-AKI

Methylation represents a key epigenetic modification involving the transfer of methyl groups to target molecules via methyltransferase catalysis. This process regulates gene expression through DNA methylation, RNA methylation, and protein methylation—the latter comprising both histone and non-histone modifications (Lister et al., 2008; Kawashima and Berger, 2014). The significance of methylation in sepsis has been acknowledged, with research indicating that sepsis can lead to changes in the methylation status of specific transcription factors, thereby influencing the inflammatory response and the degree of renal injury. Genome-wide DNA methylation analysis reveals that LPS induces abnormal methylation, primarily altering the methylation status of specific transcription factors, thereby influencing the magnitude of inflammatory responses and the severity of kidney injury (Stasi et al., 2025).

The lysine 27 of histone H3 (H3K27) is methylated by the enzyme enhancer of zeste homolog 2 (EZH2), resulting in the transcriptional silencing of target genes. Emerging evidence indicates that EZH2 is upregulated in sepsis-induced AKI. Silencing EZH2 can alleviate tubular damage by reducing apoptosis and inflammatory responses in renal tubular epithelial cells. In EZH2 knockout mice, there is a marked reduction in renal inflammation and macrophage infiltration, demonstrating that the downregulation of EZH2 participates in sepsis-induced AKI by upregulating the transcription of sex determining region Y box9 (Sox9) (Li B. et al., 2023). Targeting the EZH2/Sox9 signaling pathway may offer novel strategies for preventing or ameliorating sepsis-induced AKI. Previous studies have shown that DEX can regulate histone methylation to control excessive inflammatory responses, thereby preventing organ failure induced by sepsis. However, in LPS-induced AKI mice, DEX can effectively mitigate sepsis-induced renal failure by inhibiting the NF-κB-mediated demethylase KDM5A (Liu Y. et al., 2019). A study has investigated the role of N7-methylguanosine (m7G) methylation in SA-AKI, focusing on the regulation of methyltransferase-like 1 (METTL1), the “writer” enzyme for m7G. The findings reveal that in a sepsis model, the expression of METTL1 is significantly upregulated, promoting pyroptosis in renal tubular epithelial cells. This enhancement in stability and expression level of METTL1 further exacerbates the renal damage caused by sepsis (Wang L. et al., 2025). Additionally, MTHF2 catalyzes the m6A methylation of LX mRNA, increasing its stability and protein expression. The upregulation of LX, in turn, facilitates the progression of renal interstitial fibrosis, aggravating the symptoms of sepsis-induced AKI (Sun et al., 2025). SU3 also stabilizes TIFA mRNA through m5C methylation, and the activated TIFA subsequently initiates the NF-κB pathway, leading to an intensified renal inflammatory response and worsening the condition of sepsis-induced AKI (Zhang et al., 2025). METTL14 promotes ferroptosis in SA-AKI by increasing the m6A methylation modification of LPCAT3. During this process, the elevated m6A methylation level of LPCAT3 mRNA facilitates lipid peroxidation and the occurrence of ferroptosis by altering membrane lipid composition, thereby causing further damage to the kidney (Xu L. et al., 2025).

It is worth noting that several studies have explored methods to alleviate SA-AKI. For instance, exosomes derived from bone marrow stem cells, loaded with curcumin, have been found to mitigate SA-AKI by modulating the m6A methylation of XSR1. Specifically, exosomes enriched with the m6A demethylase FT can remove m6A modifications from XSR1 mRNA, thereby reducing its expression and subsequently decreasing p38 MAPK-mediated renal inflammation and injury (Yang et al., 2025). Furthermore, research has identified a set of kidney-specific cell-free methylation markers, such as postmeiotic segregation increased 2-like 2 (PMS2L2) recombinant tissue inhibitors of metalloproteinase 2 (TIMP2), which can be utilized to monitor the occurrence and progression of SA-AKI, offering new possibilities for early diagnosis and treatment (Zhang et al., 2023; Xu et al., 2024; You et al., 2024).

Overall, Methylation modification exerts dual effects in SA-AKI pathogenesis: on one hand, certain methylation events may exacerbate disease progression, such as by promoting pyroptosis, inflammatory responses, or ferroptosis; on the other hand, there are also methylation regulatory strategies that show potential protective effects, such as reducing inflammation and damage by inhibiting specific pathways. Elucidating these complex methylation networks advances our understanding of SA-AKI pathophysiology while offering critical insights for developing novel therapeutic interventions.

## The role of post-translational modifications in the transition from SA-AKI to CKD

Patients who survive AKI face a significantly increased risk of progressing to chronic kidney disease (CKD), imposing a substantial economic burden on both individuals and society (Wald et al., 2009). Furthermore, in critically ill patients, the co-occurrence of CKD and AKI is alarmingly prevalent, this combination is not only closely linked to delayed renal recovery but also considerably elevates the rates of rehospitalization and the incidence of end-stage renal disease (ESRD) (Abdalrahim et al., 2020). Currently, clinical strategies to prevent the progression of AKI to CKD remain unclear, suggesting that PTMs could emerge as a crucial intervention strategy in this transition. Cellular senescence refers to a state of permanent cell cycle arrest, accompanied by alterations in cell morphology, gene expression, and function. In AKI, senescence primarily affects renal tubular epithelial cells (RTECs), which are critical for normal kidney function (Hejazian et al., 2024). Senescent RTECs secrete a range of cytokines, chemoattractants, growth factors, and proteases, collectively termed the senescence-associated secretory phenotype (SASP). SASP can influence surrounding cells and the tissue microenvironment, leading to inflammation, fibrosis, and renal dysfunction (Chen et al., 2024). Growing evidence indicates that cellular senescence plays a pivotal role in the transition from AKI to CKD. Following AKI, a subset of RTECs undergoes senescence and persists long-term in renal tissue. These senescent cells continuously release SASP, promoting chronic inflammation and fibrosis, ultimately contributing to CKD progression (Rayego-Mateos et al., 2022; Puri et al., 2024). During cellular senescence, changes in PTMs can modulate the expression of senescence-associated genes, cell cycle regulation, DNA damage repair, and inflammatory responses (Xu M. et al., 2025; Grillari et al., 2010). Stasi et al. demonstrated that mTOR inhibition alleviates LPS-induced AKI by reversing the epigenetic signatures of cellular senescence in RTECs, thereby linking DNA methylation to senescence and repair mechanisms (Stasi et al., 2025). Renal tissue fibrosis is a significant hallmark of CKD, and an animal study has revealed that Dex mitigates LPS or transforming growth factor-beta 1 (TGF-β1)-induced epithelial-to-mesenchymal transition (EMT), while also preventing necrosis, necroptotic apoptosis, and pyroptosis in HK-2 cells responding to LPS stimulation (Sun Q. et al., 2024). The anti-EMT effect of DEX is associated with JNK phosphorylation (Sakai et al., 2004). Kidney fibrosis is characterized by persistent inflammation, which includes the infiltration of inflammatory cells and the secretion of cytokines (Allinovi et al., 2018). HAT and HDAC serve as enzymes for acetylation and deacetylation, playing significant roles in the progression from AKI to CKD through the regulation of acetylation status. The degree of histone H3 acetylation (H3K9Ac) is significantly upregulated in ischemic kidneys, correlating with progressive increases in MCP-1, TNF-α, TGF-β1, and collagen (Lai et al., 2019). In a cisplatin-induced AKI mouse model, low-dose cisplatin (RLDC) downregulated SIRT1 and activated NF-κB, triggering chronic renal tubular injury, tubulointerstitial inflammation, and fibrosis. Mechanistically, p53 inhibits SIRT1 to increase p65 acetylation of NF-κB activation, resulting in chronic kidney inflammation (Fu et al., 2022). Previous studies have also indicated that SIRT1 mitigates SA-AKI, particularly highlighting the SIRT1-HMGB1 signaling pathway as a key mechanism in the development of SA-AKI (Wei et al., 2019). Pan-HDAC inhibitors, such as trichostatin A (TSA) have been reported to markedly reduce renal fibrosis and improve early renal function following injury (Cianciolo Cosentino et al., 2013). Therefore, there is a compelling reason to believe that acetylation modifications play a crucial role in the transition from SA-AKI to CKD. RTECs exhibit metabolic reprogramming in the early stages of SA-AKI, and similar metabolic reprogramming occurs during the progression from AKI to CKD, leading to the pathological accumulation of lactate in the adverse microenvironment surrounding the tubular cells. The glycolytic enzyme PFKFB3 drives renal fibrosis by promoting histone lactylation-mediated activation of the NF-κB family (Wang et al., 2024b). However, current animal studies on AKI-to-CKD transition predominantly focus on ischemia-reperfusion injury (IRI) and unilateral ureteral obstruction (UOO) models, with comparatively fewer studies focusing on SA-AKI-related models. Given the bidirectional relationship between cell metabolism and histone PTMs, previous studies have primarily explored how PTMs drive metabolic reprogramming by influencing the expression of metabolic enzymes. In the transition from SA-AKI to CKD, the pathological accumulation of metabolic intermediates, such as lactate and succinate, raises questions about whether these PTMs impact renal tubular cells, leading to impaired repair and interstitial fibrosis. However, the specific sites of these PTMs modifications and the underlying mechanisms remain to be elucidated.

## Development of therapeutic strategies based on post-translational modifications

Given the unclear pathophysiology of SA-AKI, making its treatment a persistent challenge in clinical research. Currently, early intervention for SA-AKI primarily emphasizes the prompt identification of sepsis, followed by immediate therapeutic measures targeting the condition (Selby et al., 2019). These include antibiotic therapy, fluid resuscitation, hemodynamic support, immunomodulation, and organ support strategies (Evans et al., 2021). By timely addressing these critical factors, it is possible to enhance patient outcomes and mitigate the risk of complications. As sepsis is a significant risk factor for AKI, closely monitoring changes in serum creatinine and urine output in septic patients is crucial for the early detection of AKI (Peerapornratana et al., 2019). To date, there remains controversy regarding the timing of initiating dialysis, the appropriate dosage, and the choice of dialysis modality for patients with sepsis. Although some studies indicate that early initiation of renal replacement therapy is beneficial, findings in recent years have shown inconsistent conclusions across various investigations (Gaudry et al., 2016; W et al., 2015). This therapeutic uncertainty underscores the need for novel approaches. PTMs have gained attention as fundamental regulators of protein function, exhibiting remarkable diversity and disease relevance. Research have established that phosphorylation, acetylation, methylation, lactylation, and ubiquitination play vital roles in numerous biological and physiological processes. As outlined in this review, an increasing number of researchers are beginning to explore the potential significance of these modifications in the treatment of SA-AKI. This burgeoning interest offers valuable and innovative research perspectives on the pathophysiology of sepsis and its associated multi-organ dysfunction, as well as targeting therapy.

Despite extensive research on SA-AKI, effective therapeutic interventions to prevent AKI progression and improve prognosis remain elusive. This challenge may partly stem from difficulties in accurately identifying high-risk populations and achieving early diagnosis during the initial stages of kidney damage, where timely intervention is crucial for therapeutic efficacy. The conventional diagnosis of AKI relies on elevated serum creatinine (sCr) levels and/or reduced urine output, irrespective of underlying etiology, pathophysiology, or anatomical site of injury. While sCr and urine output serve as functional renal markers, they exhibit profound limitations (Wen and Parikh, 2021). Furthermore, mechanistic investigations of SA-AKI reveal discrepancies in AKI definitions between clinical and preclinical studies. Beyond traditional diagnostic criteria, preclinical research frequently incorporates renal injury biomarkers—a disparity that may represent a major translational barrier (Fiorentino et al., 2017; Fiorentino and Kellum, 2018). PTMs participate in AKI pathophysiology through multiple pathways, including inflammation regulation, immune response modulation, cell death control, metabolism regulation, and signal transduction. Consequently, PTMs-derived products hold significant potential as SA-AKI biomarkers. Mass spectrometry-based PTMs profiling of serum or tissue samples from septic patients can identify disease-associated modification sites and patterns (Miao et al., 2021; Fuller et al., 2024). Although this approach has shown progress in oncology research, its application to SA-AKI—given the condition’s high heterogeneity—may require stringent patient stratification to minimize interindividual variability (Wen and Parikh, 2021; Kumari et al., 2023). Direct PTMs products also exhibit biomarker potential. Microbial infections stimulate extracellular trap (ETs) formation in neutrophils/macrophages/monocytes, leading to citrullinated histone H3 (CitH3) release. Clinical studies demonstrate elevated CitH3 levels in septic shock patients, with serum concentrations correlating with disease severity and outcomes (Tian et al., 2021). Renal-specific cell-free DNA methylation markers enable noninvasive real-time monitoring of SA-AKI progression through circulating epigenetic signatures (You et al., 2024). NOP2/Sun RNA methyltransferase 3 (NSUN3) plays a critical role in SA-AKI. NSUN3 knockdown attenuates LPS-induced HK-2 cell damage. Mechanistically, NSUN3 stabilizes forkhead-associated domain-interacting protein (TIFA) mRNA via m5C modification, upregulating its expression—suggesting NSUN3 as a potential SA-AKI biomarker (Zhang et al., 2025)). While DNA methylation signatures show promise, tissue- and disease-specific variability in methylation patterns, along with crosstalk with other epigenetic modifications, may limit their specificity and clinical translatability (Liu et al., 2025). Although PTMs hold substantial diagnostic potential for sepsis, their detection and quantification present technical challenges. Future studies should employ high-sensitivity/high-resolution analytical methods, integrate multi-omics data to construct PTMs regulatory networks, and conduct large-scale clinical validation to elucidate PTMs mechanisms and establish their diagnostic utility in sepsis.

In fact, the development of targeted PTMs for SA-AKI is actively underway (Table 2). The class IIa HDAC inhibitor TMP195, for example, shows potential for restoring cellular function and reducing AKI severity (Zhang et al., 2020). Future investigations will likely prioritize inhibitors targeting specific phosphorylation or ubiquitination enzymes in SA-AKI. The development of novel biomarkers through the detection of specific PTMs for the early diagnosis and prognostic evaluation of SA-AKI could assist clinicians in better understanding disease progression and formulating personalized treatment plans. For example, phosphorylated MYL12B is considered a potential biomarker in the plasma of SA-AKI patients (Wu et al., 2015). Gene therapy is viewed as one of the most promising innovative treatments. Utilizing gene editing technologies such as CRISPR-Cas9 to target and regulate enzymes associated with PTMs may offer new avenues for the treatment of SA-AKI (Wu et al., 2020). In fact, there have been reports of utilizing gene editing technologies for treatment in sepsis (Rungratanawanich et al., 2023). However, Finally, due to the high heterogeneity of SA-AKI, it is possible to select the most appropriate drugs or therapeutic approaches based on the patient’s protein modification profile. By integrating specific characteristics of PTMs, personalized treatment plans can be developed to enhance the success rate of therapies. By conducting in-depth research on the mechanisms of PTMs and integrating modern drug development technologies, new strategies can be developed for the treatment of SA-AKI. In future research, interventions targeting PTMs may become a key approach to improving the prognosis of patients with SA-AKI. Advances in this field enhances our understanding of disease mechanisms while deepening our knowledge of the specific roles that PTMs play in SA-AKI through basic research. Translating these insights into clinical practice could enhance therapeutic efficacy and inform novel treatment approaches.

**TABLE 2:** Selected drugs or compounds targeting PTMs related to SA-AKI.

PTMs	Therapeutic agent	Species	Mechanism	References
Phosphorylation	MLR-1023	Mice, BUMPT	Lyn agonist MLR-1023 pretreatment improved renal function, inhibited STAT3 phosphorylation and decreased cell apoptosis.	Li et al. (2023a)
	ETBr	Mice, HK-2 cells	Inhibiting mtDNA replication by Ethidium Bromide (EtBr) treatment reduced cytosolic mtDNA accumulation and downregulated the cGAS-STING-NLRP3 axis, ameliorating the cytotoxicity.	Luo et al. (2024)
	Paeonol	Mice, dendritic cells	Paeonol inhibits the expression of phosphorylated NF-κB p65, IκBα and IKKβ, and restrains NF-κB p65 DNA-binding activity. Paeonol treatment attenuates the effects of LPS on dendritic cells, with significant inhibition of pro-inflammatory cytokines release, then TLR4 expression and NF-κB signal pathway have been suppressed.	Fan et al. (2016)
	Fortunellin	Mice, HK- cells	Fortunellin ameliorates inflammation and oxidative stress in sepsis-induced AKI, possibly through the modulation of the TLR4/NF-κB pathway.	Liu et al. (2024a)
	Embelin	Mice, BMDMs	Embelin attenuated LPS-induced septic AKI by suppressing NF-κB p65 at ser536 in activated macrophages.	Tang et al. (2023)
	UDCA	Mice, HK- cells	UDCA exerts protective effects against sepsis-induced AKI by attenuating oxidative stress and inflammation, primarily through the activation of the Nrf2/HO-1 pathway and inhibition of the NF-κB pathway.	Lou et al. (2025)
	Dihydromyricetin	Mice	DMY prevented LPS-induced AKI by increasing antioxidant capacity, reducing inflammatory responses, and blocking apoptosis.	Yin et al. (2024)
	Calpastatin	Mice, PMECs	Endothelial calpain plays a protective role in LPS-induced AKI by inhibiting p38 phosphorylation, thus attenuating iNOS expression and further decreasing NO and ROS overproduction-induced endothelial apoptosis.	Liu et al. (2020)
	H_2_S	Mice	H_2_S alleviated sepsis-induced acute kidney injury by inhibiting PERK/Bax-Bcl2 pathway.	Song et al. (2024b)
	CCT007093	Mice, HK- cells	WIP1-mediated regulation of p38 MAPK signaling attenuates pyroptosis in sepsis-associated acute kidney injury.	Wang et al. (2024a)
	MicroRNA-17-5p	Mice	miR-17-5p overexpression may exhibit a beneficial effect by attenuating LPS-induced inflammation and apoptosis via regulating the TGFβR2/TGF-β/Smad3 signaling pathway.	Sun et al. (2024a)
	2-D G	Mice, Neutrophils	2-DG reversed the downregulation of chemokine receptor 2 (CXCR2) and the impaired chemotaxis induced by CLP in mice or lipopolysaccharides (LPS) in human neutrophils.	Tan et al. (2020)
	2-D G	Mice, HK-2 cells	2‑DG treatments increased the expression of SIRT3 and phosphorylation‑AMP‑activated protein kinase (p‑AMPK), following promoted autophagy and attenuated apoptosis of tubular epithelial cells in septic mice and in LPS‑treated HK‑2 cells.	Tan et al. (2021)
Acetylation	Resveratrol	Rats, RTECs	Restored SIRT1/3 activity, reduced acetylated SOD2 levels, ameliorated oxidative stress and mitochondrial function of RTECs.	Xu et al. (2016)
	AKG2	Mice, HK-2 cells	SIRT2 inhibition increased FOXO1 acetylation, inducing its nuclear-to-cytoplasmic translocation, which promoted kidney autophagy and alleviated SAKI.	Yu et al. (2025)
	SIRT1	Mice, HK-2 cells	SIRT1 can physically interact with HMGB1 at the deacetylated lysine sites K28, K29, and K30, subsequently suppressing inflammatory signaling.	Wei et al. (2019)
	Melatonin	Mice, HK-2 cells	The protective effect of melatonin on the progression of SAKI depends on SIRT3 activation as well as mitophagic flux.TFAM-K154 site deacetylation via SIRT3 is indispensable for melatonin-enhanced mitophagic flux on SAKI.	Deng et al. (2024)
	Resveratrol/polydatin	Mice, HK-2 cells	SIRT1 attenuates sepsis-induced acute kidney injury via Beclin1 deacetylation-mediated autophagy activation.	Deng et al. (2021)
	Resveratrol/quercetin	Mice, HK-2 cells	Resveratrol/quercetin -induced activation of the deacetylase Sirt1 or the mutation of the acetylated lysine site in p53, promoted RTEC autophagy and alleviated SAKI.	Sun et al. (2021)
	EP300	NRK-52E cells	EP300-mediated H3 acetylation elevates MTHFD2 expression to reduce mitochondrial dysfunction in lipopolysaccharide-induced tubular epithelial cells.	Hu (2024)
	TMP195	Mice	TMP195 has a powerful renoprotective effect in SA-AKI by mitigating renal tubular cell apoptosis and inflammation.	Zhang et al. (2020)
	Dexmedetomidine	Mice	DEX reduces sepsis-induced AKI by decreasing TNF-α and MCP-1 and increasing BMP-7, which is associated with decreasing HDAC2 and HDAC5, as well as increasing acetyl histone H3.	Hsing et al. (2012)
	Astragaloside IV	Rats, HK-2 cells	AS-IV treatment inhibited the pyroptosis occurrence in LPS stimulated HK-2 cells and rats, related to the SIRT1 mediated deacetylation of FOXO3a.	Zha et al. (2024)
	Zn^2+^	Rats, HK-2 cells	Zn^2+^ upregulated Parkin acetylation by repressing sirtuin 7 activity to promote mitophagy and inhibit NLRP3 inflammasome activation and pyroptosis, thus improving sepsis-induced acute kidney injury.	Guo et al. (2024)
Lactylation	Glycyrrhizin/oxamate	Rats, HK-2 cells	Reversed the upregulation of NETs induced by lactate and low-dose LPS in both the blood and polymorphonuclear neutrophils (PMNs) cell supernatant, thereby ameliorating AKI associated with lactate accumulation.	Zhu et al. (2024)
Methylation	Rapamycin	Mice, RPTECs	Rapamycin treatment significantly reduced creatinine levels, preserved renal parenchyma, and counteracted the endothelial-to-mesenchymal transition (EndMT) by inhibiting the ERK pathway.	Stasi et al. (2025)
	3-deazaneplanocin A	Mice, HK-2 cells	Silencing EZH2 can protect renal function by relieving transcriptional inhibition of Sox9, activating the Wnt/β-catenin pathway, and attenuating tubular epithelial apoptosis and inflammatory response of the renal interstitium.	Li et al. (2023b)
	Dexmedetomidine	Mice	DEX could attenuate AKI through KDM5A inhibition in sepsis, transcription and protein levels of genes such as TLR4, MYD88, MTA1, PTGS2, CASP3 associated with NF-κB signaling pathway were all compromising after treated with DEX.	Liu et al. (2019b)
	Curcumin-induced exosomal FTO	Mice, HK-2 cells	Curcumin-induced exosomal FTO from bone marrow stem cells alleviates sepsis-associated acute kidney injury by modulating the m6A methylation of OXSR1.	Yang et al. (2025)
	PMS2L2	Human, CIHP-1 cells	LncRNA PMS2L2 Is Downregulated in Sepsis-Induced Acute Kidney Injury and Inhibits LPS-Induced Apoptosis of Podocytes.	Zhang et al. (2023)
Ubiquitination	Recombinant TIMP2	Mice, HK-2 cells	TIMP2 protects against sepsis-associated acute kidney injury by cAMP/NLRP3 axis-mediated pyroptosis.	Xu et al. (2024)

## Summary and perspectives

In recent decades, significant advancements have been made in understanding the diversity of PTMs and their roles in organizational homeostasis and disease (Vu et al., 2018; Liu et al., 2023). This is partly attributable to the continuous development and refinement of high-throughput omics technologies, advanced mass spectrometry techniques, and biotechnological tools, which enable us to characterize macromolecules with greater precision (Qin et al., 2022; Aizat et al., 2018; Yu et al., 2021). Nevertheless, we have only begun to unravel the complexity and intricacies of PTMs; deciphering the elaborate world of protein modifications within the human body will require relentless effort. A key challenge persist in elucidating the interactions among various PTMs and their combinatorial effects in states of health and disease. Furthermore, the reversibility of most PTMs, along with the relatively low abundance of certain modifications, presents challenges for researchers aiming to accurately capture the state of proteins under both physiological and pathological conditions (Danielson et al., 2009). These challenges must be taken into account when selecting research methodologies, and the development of techniques that enable *in situ* assessment of PTMs remains a highly anticipated technological advancement.

The relationship between sepsis and AKI involves complex biological mechanisms that remain incompletely understood (White et al., 2023; Chang et al., 2022). With the deepening research into sepsis and AKI, particularly in the realms of biomarkers, therapeutic strategies, and PTMs, we have gained a clearer understanding of the connections between the two (Peerapornratana et al., 2019; Molinari et al., 2022). However, further research is needed to uncover the specific mechanisms involved, which will provide more effective interventions and treatment options for clinical practice. We anticipate that the translationally modified derivatives may exhibit significant therapeutic potential in SA-AKI, akin to high-density lipoprotein (HDL). In sepsis patients, HDL levels are markedly reduced and correlate with poor prognosis (Cao et al., 2022). Animal models have similarly demonstrated the crucial role of HDL in modulating inflammatory and immune responses. HDL attenuates inflammatory reactions by regulating the expression of adhesion molecules on leukocytes and endothelial cells, thereby inhibiting leukocyte adhesion, rolling, and tissue infiltration. Furthermore, HDL modulates immune function by altering cholesterol content in the plasma membrane of immune cells, influencing membrane fluidity and signal transduction, which subsequently suppresses the activation of key immune receptors (Stasi et al., 2021). Endothelial dysfunction represents a pivotal event in the pathophysiology of sepsis, contributing to increased vascular permeability, impaired vasodilation, and coagulation abnormalities (Vincent et al., 2021). HDL exerts endothelial protective effects through multiple mechanisms, including promoting nitric oxide (NO) production, suppressing endothelial inflammation, and exerting antioxidant activity (Lin et al., 2024; Tanaka et al., 2020; Joffre and Hellman, 2021). Building upon promising preclinical findings, clinical translational studies on HDL have further demonstrated that recombinant CER-001 high-density lipoprotein (rHDL) can provide anti-inflammatory and endothelial protective effects, preserve renal and hepatic integrity and function, reduce the risk of AKI, and improve clinical outcomes. Future research efforts should focus on establishing more systematic assessment and management strategies for the relationship between sepsis and AKI, with the goal of improving patient survival rates and quality of life. Studying the changes and mechanisms of these modifications will provide new insights into the pathophysiology of AKI and may serve as a foundation for developing novel diagnostic and therapeutic approaches. As research advances, PTMs are expected to emerge as important targets for the treatment of SA-AKI, thereby promoting the development of clinical applications.
