# Correction: Post-translational modifications in sepsis-induced acute kidney injury: mechanisms and perspectives

**DOI:** 10.3389/fphar.2025.1710281

**Published:** 2025-10-10

**Authors:** Lin Song, Wei Jiang, Ke Liu, Jing Wang, Weilei Gong, Jiangquan Yu, Ruiqiang Zheng

**Affiliations:** ^1^ Northern Jiangsu People’s Hospital Affiliated to Yangzhou University, Yangzhou, China; ^2^ Intensive Care Unit, Northern Jiangsu People’s Hospital, Yangzhou, China; ^3^ Yangzhou University Hospital, Yangzhou, China; ^4^ School of Pharmaceutical Sciences and Institute of Materia Medica, Shandong First Medical University and Shandong Academy of Medical Sciences, Jinan, China

**Keywords:** acute kidney injury, sepsis, post-translational modifications, sepsis-induced acute, kidney injury, inflammation

There was a mistake in the order of the Figures 2–5 as published. The figures and their corresponding figure captions for these results were all misaligned. The corrected figures and their captions appear below.

**FIGURE 2:**
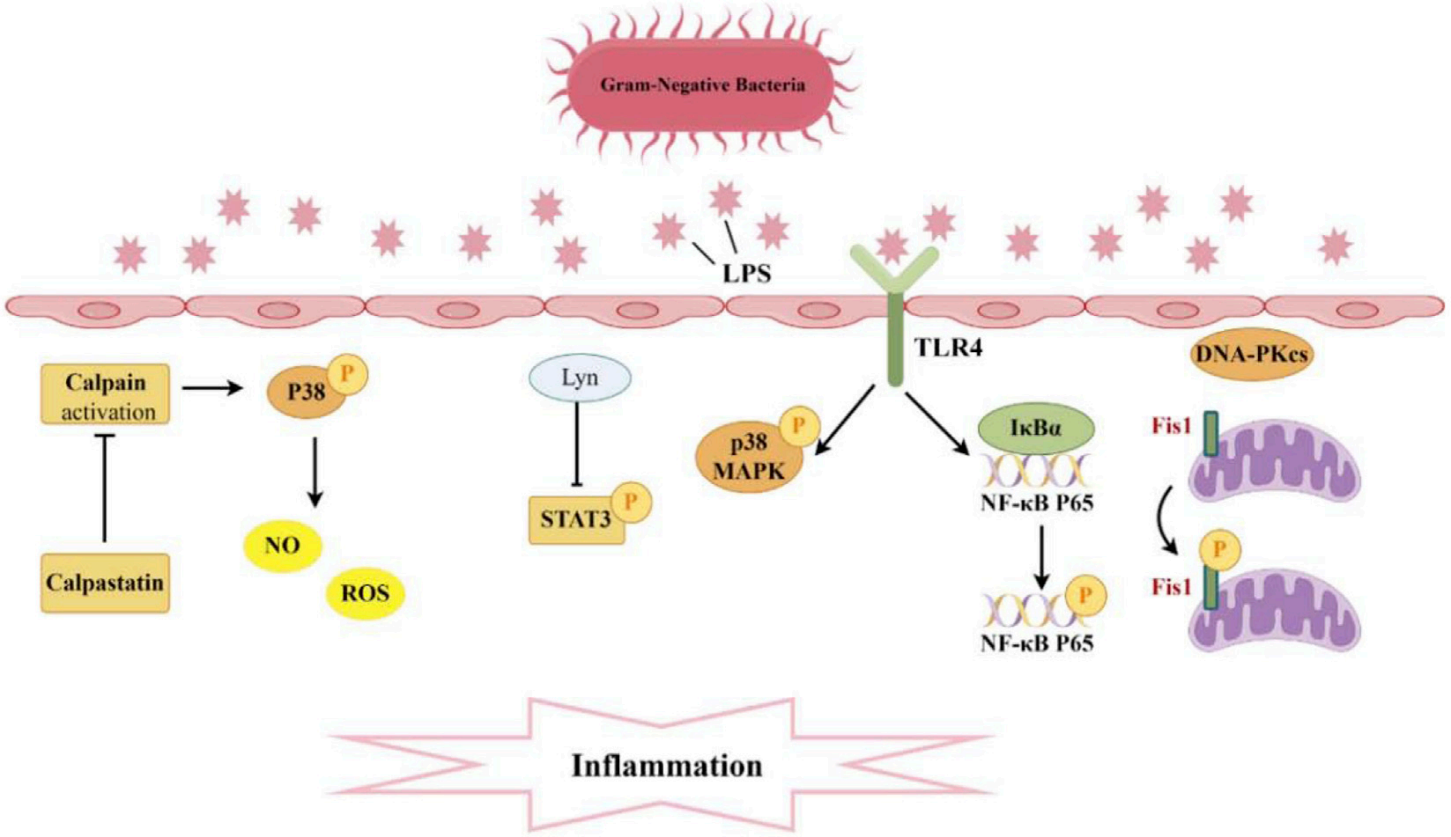
Pathogenesis associated with Phosphorylation and sepsis-induced acute kidney injury. A significant production of inflammatory cytokines occurs within the glomeruli and renal tubular interstitium. The interaction between TLR4 and LPS activates the phosphorylation of P38 MAPK and NF-κB, while Lyn inhibits the phosphorylation of STAT3, thereby diminishing levels of inflammatory mediators. Moreover, the suppression of calpain activation can curtail P38 phosphorylation, reduce ROS, and consequently mitigate endothelial cell apoptosis. Additionally, DNA-PKcs can induce the phosphorylation of Fis1, resulting in mitochondrial dysfunction and subsequent cell apoptosis.

**FIGURE 3:**
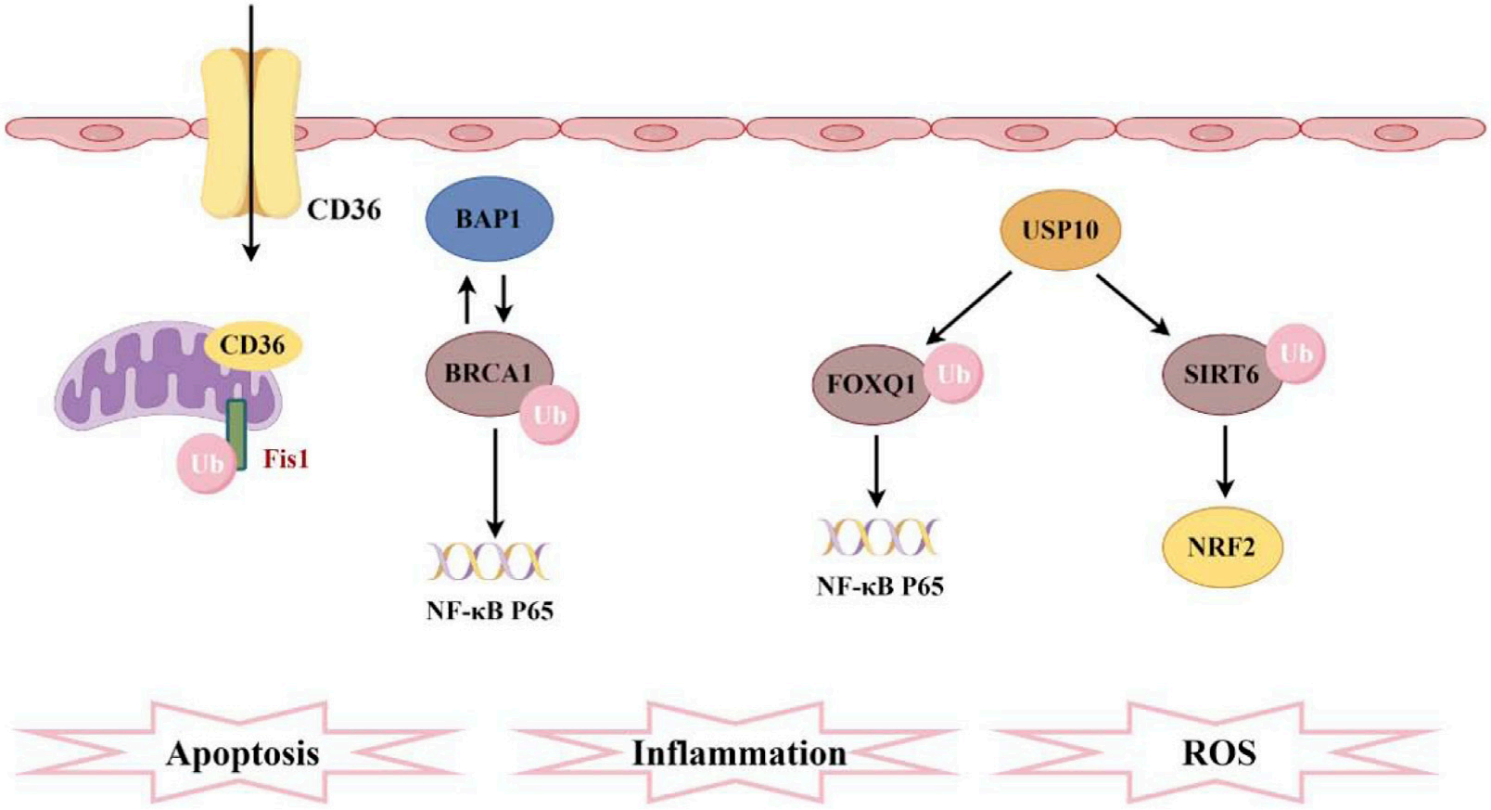
Pathogenesis associated with ubiquitination and sepsis-induced acute kidney injury. CD36 promotes ferroptosis in proximal tubular cells by regulating the ubiquitination of FSP1. The interaction between BAP1 and BRCA1 enhances the stability of BRCA1 protein through deubiquitination, thereby inhibiting NF-κB. Furthermore, FOXQ1, deubiquitinated by USP10, ameliorates cellular inflammation and apoptosis. Additionally, USP10 interacts with SIRT6 to suppress its ubiquitination, alleviating oxidative stress.

**FIGURE 4:**
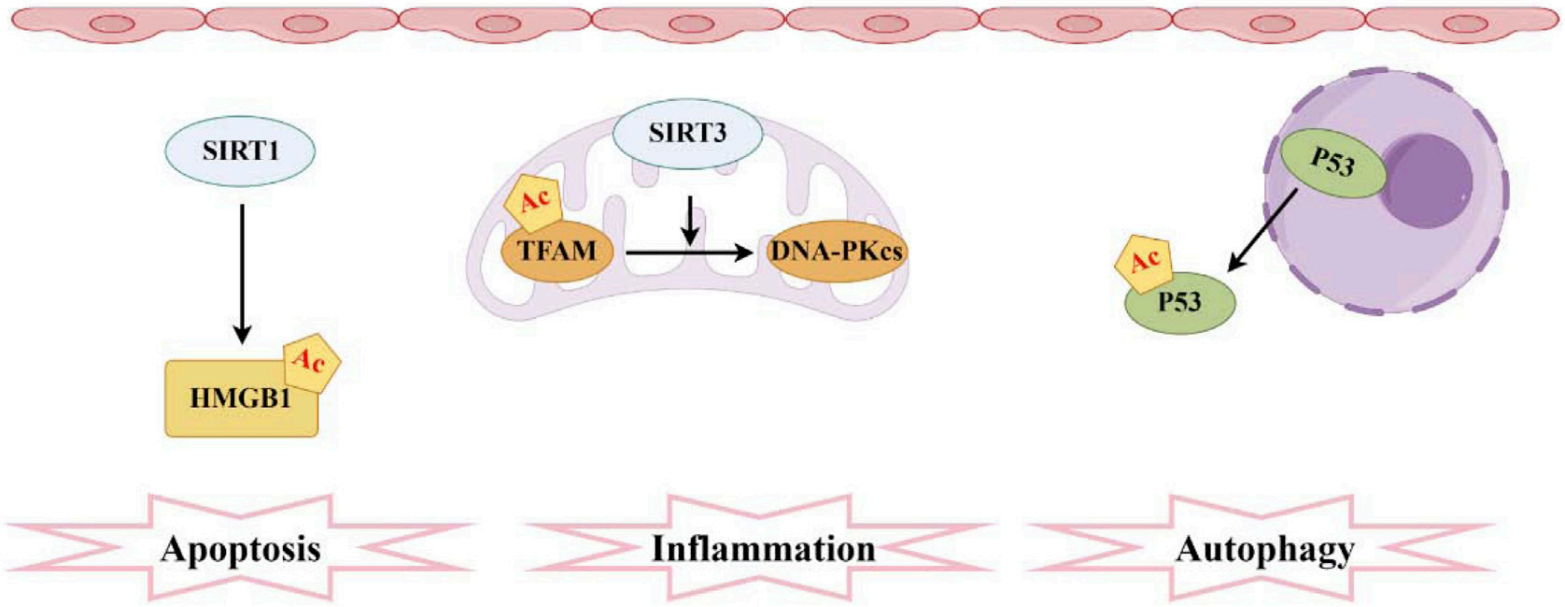
Pathogenesis associated with acetylation and sepsis-induced acute kidney injury. The sirtuin family comprises the most prevalent deacetylases, with SIRT1 mediating the acetylation of HMGB1 and SIRT3 facilitating the acetylation of TFAM. Moreover, elevated levels of acetylated P53 in RTECs hinder autophagy.

**FIGURE 5:**
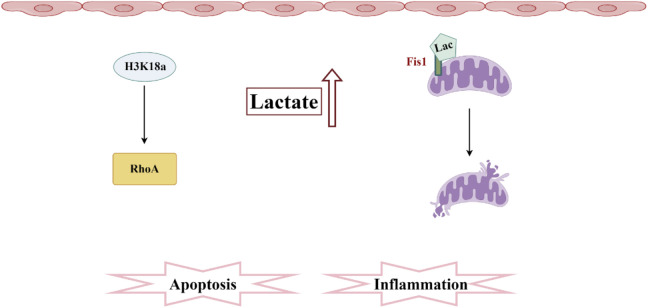
Pathogenesis associated with lactylation and sepsis-induced acute kidney injury. In SA-AKI, elevated levels of lactate and histone lactylation, particularly the increased lactylation of H3K18, activate RhoA protein, thereby triggering inflammation and apoptosis. Additionally, lactate mediates the lactylation of Fis1, promoting mitochondrial fission and exacerbating cellular apoptosis.

There was a mistake in the caption of Figure 5 as published. Instead of “Pathogenesis associated with Ubiquitination and sepsis-induced acute kidney injury” it should be “Pathogenesis associated with lactylation and sepsis-induced acute kidney injury”. The corrected caption of Figure 5 appears below.

The original article has been updated.

